# Cost minimization analysis of nasopharyngoscope reprocessing in community practice

**DOI:** 10.1186/s40463-022-00610-9

**Published:** 2023-02-08

**Authors:** Ameen Biadsee, Lauren Crosby, Winsion Chow, Leigh J Sowerby

**Affiliations:** 1 Department of Otolaryngology- Head and Neck Surgery, Western University, 268 Grosvenor Street, London, ON N6A 4V2 Canada; 2 Department of Otolaryngology- Head and Neck Surgery, Meir Medical Center, Kfar Saba, Israel; 3 Sackler Faculty of Medicine, Tel-Aviv University, Tel-Aviv, Israel; 4 Department of Anesthesiology, Perioperative and Pain Medicine, Foothills Medical Centre, University of Calgary, Calgary, Canada; 5 Deptartment of Otolaryngology- Head and Neck Surgery, University of Ottawa, Ottawa, ON Canada

**Keywords:** Otolaryngology, Nasopharyngoscope, Decontamination, Reprocessing, Cost analysis

## Abstract

**Background:**

Reprocessing of nasopharyngoscopes represents a large financial burden to community physicians. The aim of this study was to perform a cost analysis of nasopharyngoscope reprocessing methods at the community level.

**Methods:**

Electronic surveys were distributed by email to community otolaryngologists. Surveys were comprised of 14 questions assessing clinic size, nasopharyngoscope volume, scope reprocessing method and maintenance. Four manual techniques were evaluated: (1) soak with ortho-phthalaldehyde solution (Cidex-OPA; Advanced Sterilization Products, Johnson and Johnson Inc., Markham, Canada), (2) soak with accelerated hydrogen peroxide solution (Revital-Ox; Steris Canada Inc., Mississauga, Canada), (3) disinfection with chlorine dioxide wipe (Tristel Trio Wipes System; Tristel plc., Cambridgeshire, UK), (4) UV-C light system (UV Smart, Delft, The Netherlands). All costs are reported in CAD, and consumable and capital costs for reprocessing methods were obtained from reported vendor prices. Time costs were derived from manufacturer recommendations, the Ontario Medical Association Physician’s Guide to Uninsured Services, and the Ontario Nurses Association Collective Agreement. Cost analyses determined the most cost-effective reprocessing method in the community setting. Sensitivity analyses assessed the impact of reprocessing volume and labour costs.

**Results:**

Thirty-six (86%) otolaryngologists responded and answered the survey. The cost per reprocessing event for Cidex-OPA, Revital-Ox, Tristel and UV system were $38.59, $26.47, $30.53, and $22.74 respectively when physicians reprocessed their endoscopes themselves. Sensitivity analyses demonstrated that Revital-Ox was the least costly option in a low volume, however, the UV system remained the most cost effective in higher volumes. The cost per reprocessing event when done by clinic staff was $5.51, $4.42, $11.23 and $6.21 for Cidex-OPA, Revital-Ox, Tristel and the UV system.

**Conclusions:**

The UV light system appears to be the most cost-effective method in high volumes of reprocessing, and Revital-Ox is cheaper in lower volumes and when performed by clinic staff rather than physicians. It is important to consider the anticipated work volume, shared clinic space and number of co-workers prior to choosing a reprocessing method.

**Graphical Abstract:**

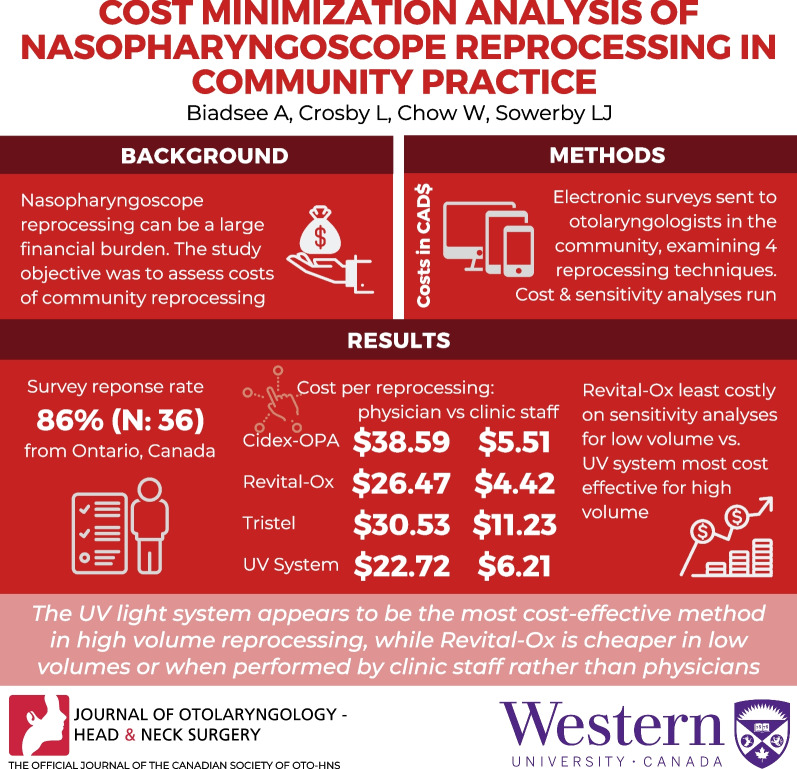

## Introduction

The nasopharyngoscope has become an indispensable tool for the physical examination of patients in the modern Otolaryngology practice. Given the large number of patients who require nasopharyngoscopy as part their head and neck exam, scopes are often reprocessed and re-used multiple times over the course of one clinic day. Therefore, reprocessing them in a manner which is efficient, low-cost, and prioritizes patient safety is of high importance.

Nasopharyngoscopes are classified as semi-critical devices, which includes any endoscope that comes into contact with mucous membranes [1]. Semi-critical endoscopes must undergo high-level disinfection (elimination of mycobacteria, bacteria, and viruses) as part of their reprocessing. This can be done manually; however, the use of automated endoscope reprocessing systems (AER) is now encouraged [1]. Sowerby and Rudmik [2] compared two AER protocols and two manual reprocessing protocols in a Canadian tertiary care setting and found that manual reprocessing with accelerated hydrogen peroxide solution (Revital-Ox; Steris Canada Inc., Mississauga, Canada) was the most cost-effective method for nasopharyngoscope decontamination. Protocols were not evaluated for lower volume settings reflective of community practice in a non-hospital setting.

It is broadly accepted in Otolaryngology—Head and Neck Surgery that the maintenance and reprocessing of nasopharyngoscopes represents a larger burden to community physicians compared to hospital-based physicians, but this has not yet been quantified. The aim of this study was to perform a cost analysis of nasopharyngoscope reprocessing methods at the community level, with the intention that conclusions will help otolaryngologists choose the highest value method for their particular setting and guide revision of provincial policy to reflect appropriate and cost-effective reprocessing practices.

## Methods

A cost-minimization analysis was performed by evaluating four nasopharyngoscope reprocessing techniques: (1) high-level decontamination with manual ortho-phthalaldehyde solution (Cidex-OPA; Advanced Sterilization Products, Johnson and Johnson Inc., Markham, Canada), (2) high-level decontamination with manual accelerated hydrogen peroxide solution (Revital-Ox; Steris Canada Inc., Mississauga, Canada), (3) high-level decontamination with manual chlorine dioxide wipe (Tristel Trio Wipes System; Tristel plc., Cambridgeshire, UK) and (4) high-level decontamination with a UV-C light system (UV Smart, Delft, The Netherlands).

A literature review was conducted to identify the appropriate reprocessing techniques to include in the cost analysis, and to compare their effectiveness. Cidex-OPA and Revital-Ox were found to be the two most common manual reprocessing techniques used in Canada [2]. The Tristel Trio Wipes System has not yet been approved in North America, but is in routine use in Oceania and Europe, and would represent a portable and convenient alternative to other manual techniques should it become available [2]. The UV-C light system is approved in Europe and the United States, and is under consideration for approval in Canada. The recommended protocol for decontamination with Cidex-OPA involves a wipe at the point of use to clean any debris, immersion in an enzyme solution to remove remaining proteins, soaking in the disinfectant solution for 12 min, followed by three consecutive 1-min rinses [3]. Decontamination with Revital-Ox follows a similar protocol, however disinfectant immersion is 5 min, followed by a 1-min rinse [4]. Decontamination with the Tristel wipes involves an enzyme wipe at the point of use, followed by a chlorine dioxide disinfecting wipe, a drying time of 30 s, and a rinsing wipe [5]. UV light decontamination process includes manually pre-cleaning the nasopharyngoscope with a soaked-wipe (with water) for 20 s, then the endoscope is exposed to UV-C light for 60 s while suspended [6].

When manufacturer recommendations are followed, all four techniques provide equivalent high-level disinfection against mycobacteria, bacteria, and viruses, therefore a cost-minimization analysis was indicated [7].

A time-driven, activity-based micro-costing approach was used for the cost analysis to ensure that costs per reprocessing event accounted for staffing requirements and capital depreciation. This approach directly allocates both labor and consumables to an event based on the time required and takes into account opportunity cost for activities. The primary outcome measure was annual reprocessing cost. Capital and consumable costs for Cidex-OPA and Revital-Ox were obtained from wholesale vendor prices through Healthcare Materials and Management Services, London, Canada and are presented in Canadian dollars as of January 2022. As Tristel wipes and the UV light system are currently not available in Canada, the cost information for these systems were obtained from Tristel and UV-Smart directly and is presented in 2022 Canadian dollars using April 21, 2022 exchange rate. (Tristel plc and UV Smart, personal communications, Written communication, January and April 2022). Time costs were derived from manufacturer reprocessing protocols [3–5], and the Ontario Medical Association (OMA) Physician’s Guide to Uninsured Services, 2019 [8] to value the opportunity cost associated with missed billable work as a result of scope reprocessing.

The base case was developed using the cost information outlined above and survey data regarding nasopharyngoscopy use in community practice. Electronic surveys were distributed by email to 42 community otolaryngologists registered through the Canadian Society of Otolaryngology—Head and Neck Surgery. Surveys were comprised of 14 questions assessing clinic size, nasopharyngoscope volume, scope reprocessing method, and maintenance. Responses were used to calculate mean reprocessing volumes per surgeon for the financial model. Generalizability of the model was assessed by analyzing survey respondents’ self-reported compliance with manufacturer recommendations for reprocessing.

Sensitivity analyses were conducted to account for the uncertainty of assumptions made by the base case scenario. Highest and lowest reported annual reprocessing volumes were used to assess the sensitivity of the primary outcome to differences in volume. Labour costs for nursing staff were used to assess the effect on the primary outcome in a scenario where non-physician clinic staff are responsible for reprocessing. Costs were derived from the Ontario Nurses Association (ONA) Collective Agreement on RN Compensation, 2021 [9].

## Results

### Survey demographics

Thirty-six (86%) completed surveys were returned, representing physicians from 7 Canadian provinces. Respondents reported that on average, each practice was comprised of 1.9 surgeons, who each worked 3 clinic days per week, and scoped 18 patients per clinic (Table 1). The average practice had 3.3 nasopharyngoscopes, with the most common types being—in order—Olympus, Pentax, and Storz. Most sites reported using Cidex-OPA for disinfection (80%), while 16% of sites used Revital-Ox. One site reported using endoscope sheaths. Surgeons were responsible for reprocessing scopes at 23 (64%) sites, while nurses, administrators, and technicians reprocessed scopes at the remaining 13 sites. The base case for the financial model was established from the survey results. In the base case, an individual surgeon would have 3 clinic days per week, scope 18 patients per clinic, reprocess their own scopes, and work 48 weeks of the year. This case results in 2,592 reprocessing events annually.

**Table 1:** Survey demographics

Questions	Mean	Range
Number of surgeons per site	1.9	1–5
Number of nasopharyngoscopes	3.3	1–9
Number of clinic days per week	2	1–5
Number of patients scoped per clinic	18	8–25

### Consumables and capital

Consumable and capital costs per reprocessing cycle are outlined in Table 2. One reprocessing cycle refers to the process of decontaminating one scope after use on a patient. For scopes soaked in a disinfecting solution, a cleaning wipe is required at the point of care after each use to remove any gross debris. Wipes (Accel) cost $10.32 for a box of 160 and can be used with Cidex-OPA, Revital-Ox and UV system. Scopes are then soaked in a 200 mL enzyme bath, which is replaced with each reprocessing cycle, to remove residual proteins. Enzyme solutions (Medzyme, emPower) cost on average $35.92 for a 4L container. Cidex-OPA is sold in 3.8L bottles, which cost $43.25, and Revital-Ox is sold in 4L bottles which cost $40.12. There is no standard size for cleaning containers and there are many different sized containers on the market. For comparison purposes, we used the Revital-Ox container. Two bottles of disinfectant are required every 14 days, regardless of reprocessing volume. Testing strips are required before each cycle to ensure the disinfectant solution is above the minimum effective concentration. Cidex-OPA strips cost $71.75 for a box of 200, while Revital-Ox strips cost $47.25 for a box of 100. Gloves are used at each step of the cycle (pre-cleaning, disinfecting, rinsing) and cost $0.224 per pair. Soaking trays are replaced annually and cost on average $196. The Tristel Trio Wipes System involves three stages of reprocessing and includes a wipe for each stage (pre-cleaning, disinfecting, and rinsing). Together, the wipes cost $9.24 per cycle. Gloves are to be changed at each step.

**Table 2:** Financial model

Consumable and capital					Time (min)				
Per cycle	Cidex-OPA	Revital-Ox	Tristel	UV system	Per cycle	Cidex-OPA	Revital-Ox	Tristel	UV system
Wipe	0.064	0.064	0	0.064	Wipe	1	1	1	2
Enzyme solution	0.1	0.1	0	0	Enzyme bath	2	2	0	0
Testing strips	0.36	0.47	0	0	Disinfection	12	5	1.5	1
Disinfectant	0.82	0.76	8.57	3.75^a^	Rinse	3	1	1	0
PPE (gloves)	0.67	0.67	0.67	0.67	Scope downtime	18	9	3.5	3
Soaking tray	0.077	0.077	0	0	Labour time	6	4	3.5	3^b^
Cost	$2.09	$2.14	$9.24	$4.5	Cost (MD)^c^	$36.50	$24.33	$21.29	$18.24

^a^Capital cost of D90 UV system and service per one cycle

^b^One minute of labour time is added for loading and tracking the endoscope

^c^From OMA Physician’s Guide to Uninsured Services, 2019

The UV light system requires the D60 unit for flexible endoscopes. The price of the D60 unit is $89,056 CAD ($70,000 USD). Additionally, it requires a yearly service and maintenance to ensure its effectiveness in decontamination, of a yearly price $8270 CAD ($6500 USD). Using gloves, a mechanical pre-cleaning with a wipe (Accel) is necessary to achieve effective decontamination. In this method there is no need for disinfectant solutions. To calculate the cost the UV system disinfection per cycle, we used a 10-year life expectancy of the system as a model (suggested by the retailer). We included the cost of the D60 system and a 10-year service fee, then calculated the cost per cycle using the average cost of the system per year divided with the base case volume.

Costs per cycle for all four techniques were determined using the base case inputs. When considering direct costs per cycle, Cidex-OPA is the least costly method at $2.09, followed by Revital-Ox at $2.14, and then UV-C light system at $4.50. Tristel was the most expensive with consumable costs of $9.24.

### Labour costs

Time associated with each step of the reprocessing cycle is described in Table 2. Labour costs are attributed to steps in the cycle in which the physician or provider is required to actively participate, including wiping, transferring, and rinsing the scopes. Due to the longer rinse time required for Cidex-OPA, it is associated with higher labour costs ($36.50) than the other methods, while the UV light system has the lowest labor costs and the shortest scope downtime, as reprocessing does not require an enzyme bath or soak with disinfectant (Table 2).

### Total costs

The total cost per reprocessing event and total annual cost for each method are outlined in Table 3. At the base case volume of 2592 reprocessing events per year, the UV system is the most cost-effective reprocessing method, at $58,942.08 per year, compared to Revital-Ox system at $68,610.24, Tristel at $79,133.76, and Cidex-OPA at $100,025.28 per year.

**Table 3:** Cost analysis

Reprocessing costs	Cidex-OPA	Revital-Ox	Tristel	UV system
Reprocessing cost per patient	$38.59	$26.47	$30.53	$22.74
Reprocessing cost per clinic day	$694.62	$476.46	$549.54	$409.32
Reprocessing cost per year	$100,025.28	$68,610.24	$79,133.76	$58,942.08
Reprocessing cost per year [excluding labour costs]	$5417.28	$5546.88	$23,950.08	$11,664

### Sensitivity analysis

Similar results were found with sensitivity analysis for volume. At the lower volume of 480 events per year, Revital-Ox system was more cost-effective, and at the higher volume of 4320 events per year, the UV-C system was the least costly method (Table 4).

**Table 4:** Sensitivity analysis: reprocessing volume

Reprocessing costs	Cidex-OPA		Revital-Ox		Tristel		UV system	
	Low volume	High volume	Low volume	High-volume	Low volume	High volume	Low volume	High volume
Cost per patient	$42.41	$38.22	$30.04	$26.11	$30.53	$30.53	$39.25	$21.22

*Low volume* lowest reported annual reprocessing volume = 480 scopes

*High volume* highest reported annual reprocessing volume = 4320 scopes

A scenario analysis for labour costs was done to compare the cost of physicians reprocessing their own scopes, to reprocessing done by other clinic staff. Nursing staff were reported to be the second most likely to reprocess scopes in the community setting. Annual reprocessing costs are significantly less when reprocessing is done by nursing staff, and this is true for all four methods (Table 5).

**Table 5:** Scenario analysis: labour costs

Reprocessing costs	Cidex-OPA		Revital-Ox		Tristel		UV system	
	Physician processing^a^	Clinic staff processing^b^	Physician processing^a^	Clinic staff processing^b^	Physician processing^a^	Clinic staff processing^b^	Physician processing^a^	Clinic staff processing^b^
Cost per patient	$38.59	$5.51	$26.47	$4.42	$30.53	$11.23	$22.74	$6.21
Cost per clinic day	$694.62	$99.18	$476.46	$79.56	$549.54	$202.14	$409.32	$111.78
Cost per year	$100,025.28	$14,281.92	$68,610.24	$11,456.64	$79,133.76	$29,108.16	$58,942.08	**$16,096.32**

^a^From OMA Physician’s Guide to Uninsured Services, 2019

^b^From ONA Collective Agreement—RN Compensation, 2021

In this scenario, UV light system was found to be the most cost-effective method for reprocessing, while Tristel was found to be the least cost-effective method, at almost twice the annual cost of the next closest alternative.

### Compliance with manufacturer recommendations

**Fig. 1:**
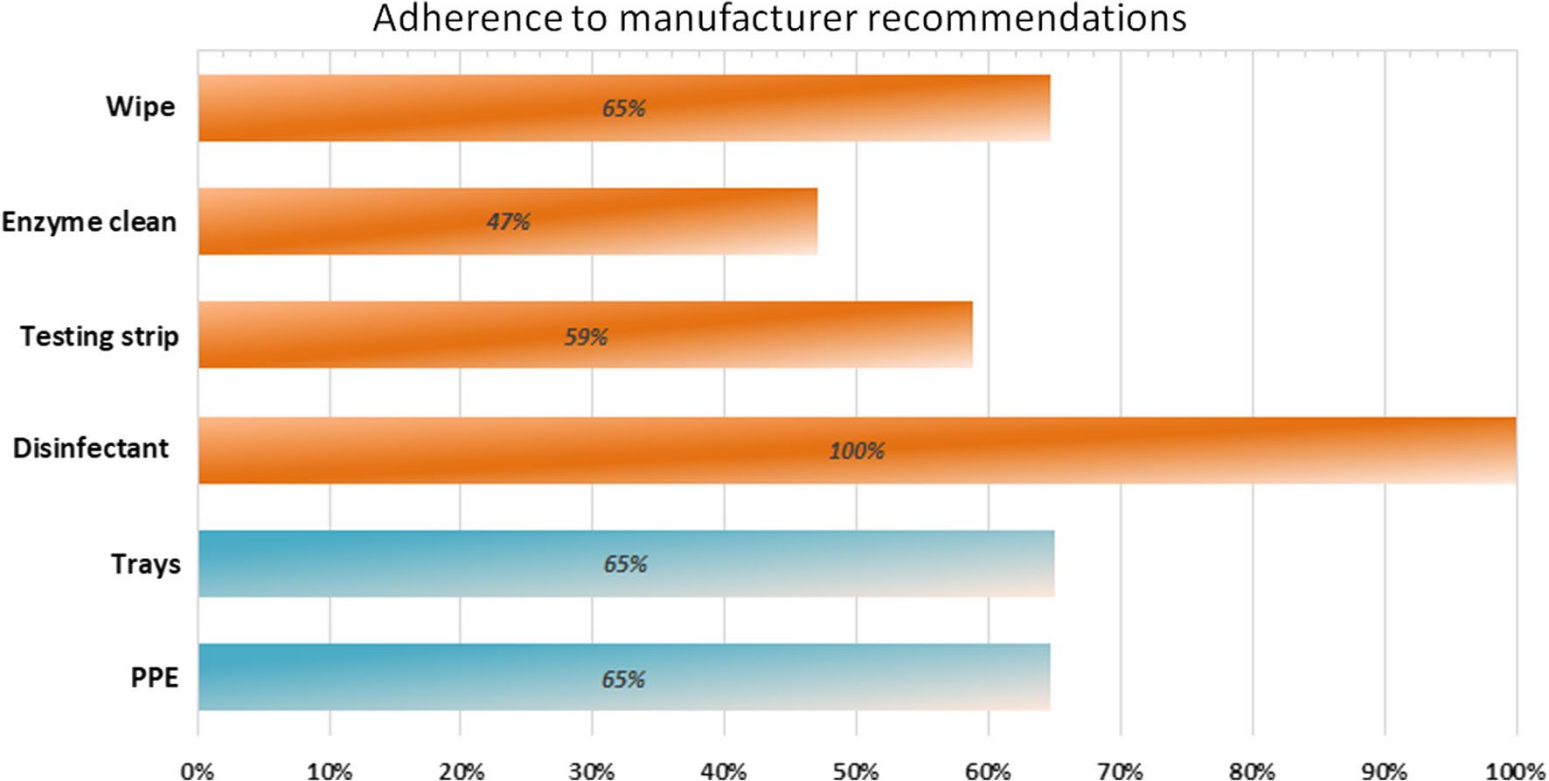
Respondent adherence to reprocessing steps recommended by the manufacturer

Survey results indicate that in practice, most physicians do not comply with manufacturer recommendations for reprocessing (Fig. 1). Adherence to each step in the reprocessing cycle is variable, ranging from 100% compliance with the disinfectant soak to 47% compliance with the enzyme clean. Only 17% of respondents reported that all recommended reprocessing steps are followed at their site. These results only apply to Cidex-OPA and Revital-Ox, as the Tristel Trio Wipes System and the UV system are not yet approved for use in Canada (Fig. 1).

### Repair costs

Complete data regarding repair requirements was not reported by all respondents. Of the 15 respondents that reported needing repairs to their endoscopes, the cost of repair was an average of $2713 ± 1847. Twelve respondents had not yet needed to repair an endoscope. Of those reporting annual replacement data, the lifespan of an endoscope was reported to range between 3 and 12 years.

## Discussion

In community settings, where manual reprocessing of nasopharyngoscopes is often the responsibility of the physician, the importance of cost-effective, efficient systems which maximize patient safety and minimize the risk of scope damage cannot be understated. This cost analysis has demonstrated that UV-C disinfection solution is the least costly reprocessing method, however it not currently available to community otolaryngologists. This is due to the higher time costs associated with the most common reprocessing methods, such as Cidex-OPA ortho-phthalaldehyde solution or Revital-Ox solution, and the higher consumable costs associated with the Tristel Trio Wipes System. This potentially holds true regardless of the reprocessing volume of the clinic, or the individual responsible for reprocessing the nasopharyngoscopes.

In interpreting these results, one must keep in mind that to realize the cost savings associated with switching reprocessing methods, or off-loading reprocessing responsibility to clinic staff, physicians would need to convert all time saved into time seeing additional patients. In theory this should be achievable given that in the average clinic day for our base case, a physician will spend 108.6 min reprocessing with Cidex-OPA, 72.4 min reprocessing with Revital-Ox, and 63.35 min reprocessing with Tristel, and 54 min of reprocessing with the UV system. Switching or off-loading should allow sufficient time for additional patient encounters in mid to high volume clinics. This may not be the case in practice however, as some survey respondents report that they reprocess their scopes at the point of care; cleaning, soaking and the rinsing scopes while they are performing other components of the patient consultation. In these cases, labour costs, which were valued as the opportunity costs of missed billable work, may be negligible.

Both reprocessing methods currently in use in Canada have been associated with implicit costs not accounted for by this financial model. Incomplete survey responses for repair and replacement costs did not allow for these parameters to be included in the model, however anecdotal evidence from three surgeons suggested that Revital-Ox is associated with higher rates of scope damage. This could increase the cost associated with this method to the extent that it surpasses Tristel and Cidex-OPA. The average cost of repairing nasopharyngoscopes reprocessed with an automated reprocessor was reported to be $327 USD for minor repairs and $3816 USD for major repairs, for an average of $2501USD, with endoscopes requiring repair approximately every 56 cycles [10]. While complete repair data was not collected in this data set, the cost for repair is in keeping with the previously reported numbers, but the incidence of damage appears to be substantially less with manual reprocessing. No data is available in the literature regarding Tristel wipes or the UV-C system and the impact on endoscope repairs, but early reports from both manufacturers indicate that there is a significant reduction in repairs. One respondent remarked that they have diverged from manufacturer reprocessing guidelines for Revital-Ox in order to minimize damage to their scopes, which could alter the effectiveness of the decontamination method to where it is less effective than the other three alternatives.

It has been generally accepted that OPA residue remains on endoscopes even after multiple rinses. In 2007, three cases of OPA-induced anaphylaxis were reported in patients undergoing serial laryngoscopy for head and neck cancer surveillance [11]. In 2015, an additional three cases were reported in otolaryngology, along with 17 reactions across other surgical specialties [12]. As a result, The Medicine and Healthcare products Regulatory Agency in the United Kingdom now recommends the use of validated alternatives to OPA [13]. Jiang et al. [14] investigated nasopharyngoscope device failure associated with contamination using data from the US Food and Drug Administration Manufacturer and User Facility Device Experience database and reported one injury due to incomplete removal of the disinfectant agent (i.e. CIDEX-OPA).

Not only are anaphylactic reactions associated with higher healthcare costs, but the potential risk of adverse events could decrease the effectiveness of Cidex-OPA to less than that of other reprocessing methods, in which case a cost minimization analysis would not be the most appropriate study design.

Another possible additional cost is the neutralization of the disinfectant solution prior to disposal. Depending on local regulations of environmental and health safety units, the use of glycine powder might be needed in order to neutralize environmentally hazardous disinfectants, like Cidex-OPA, prior to disposal. Single use disposable sterile slide-on sheath cover for flexible endoscopes were evaluated and approved by the US Food and Drug Administration [15]. These protective sheaths can provide a fast and safe method that protects patients from cross-contamination [16]. However, the risk of micro perforations unfortunately remains a reason for most provincial jurisdictions in Canada to require high-level processing even if an endoscope sheath is used.

Perhaps the most significant limitation to this analysis is the assumption that community physicians are reprocessing nasopharyngoscopes according to manufacturer recommendations. Our results indicate only 17% of physicians follow each step outlined by manufacturers. This finding is in fitting with a previous study by Brake et al. [17] which identified significant variation in protocol and methodology used for scope decontamination nationwide. Although manufacturers provide recommendations for use of their products, there is little guidance at the health system level specific to nasopharyngoscope reprocessing.

In provincial and national guidelines in Canada for infection control and prevention, nasopharyngoscopes are often grouped with other semi-critical endoscopes, including bronchoscopes, cystoscopes and colonoscopes, which have multiple lumens and enter cavities associated with higher risks of infection transmission [18–20]. Some guidelines do not mention nasopharyngoscopes at all [21, 22]. Therefore, variability in reprocessing technique among physicians is not surprising given the heterogeneity of national and provincial expectations. A possible contributor to the lack of compliance with guidelines is that by grouping nasopharyngoscopes with other endoscopes, guidelines are requiring over-processing. Higher costs and reduced efficiency associated with over-processing may be prohibitively costly and time consuming for physicians, in addition to increasing wear and tear on nasopharyngoscopes. Internationally, regulatory agencies have acknowledged the difference between nasopharyngoscopes and other flexible endoscopes, allowing the approval of novel streamlined systems for reprocessing nasopharyngoscopes. Tristel Trio Wipes System (Tristel plc), which is not yet approved in North America, condenses reprocessing into three simple, brief steps which can be performed at the point of care. It is routinely used in Europe and Australia [23] and was found by Sowerby et al. [2] to be the most cost-effective reprocessing method in low-volume settings when compared to reprocessing methods commonly used in North American hospital-based clinics. However, the UV-C system for reprocessing non-channeled endoscopes is promising and has demonstrated efficacy [6, 24]. UV-C light has been used for surface disinfection for many years and is effective against nosocomial pathogens and biofilms [25, 26].

This analysis found the UV light system to be the most cost-effective reprocessing method in the community setting in a high-volume clinic. However, Revital-Ox was more cost-effective in small volume clinics. Revital-ox or Tristel may present a convenient alternative for physicians who reprocess their own scopes in a low-volume setting. The UV-C system is the fastest method for nasopharyngoscope reprocessing, potentially allowing for fewer nasopharyngoscopes to be available in a clinic, thus reducing capital and maintenance costs. The simplicity of the system may also facilitate adherence to manufacturer recommendations.

Regardless of the reprocessing method, the burden of reprocessing nasopharyngoscopes on physicians is notably larger in the community setting than in tertiary centres, where reprocessing is often under the purview of a central processing department [2]. For this reason, nasopharyngoscopy may become a less attractive aspect of practice for community physicians, which risks having them defer patients to hospitals for this assessment. Tray fees are service benefits used by provinces such as Alberta to offset the opportunity costs of missed billable work associated with activities such as nasopharyngoscope reprocessing for community physicians [27]. Provincial authorities in other regions should consider their implementation to ensure community otolaryngologists have the means to continue providing this essential diagnostic assessment for patients in lower acuity settings, especially in areas where access to tertiary care is limited, either geographically or secondary to wait times.

## Conclusions

By applying a cost-minimization analysis to a base case scenario informed by general practice otolaryngologists, four nasopharyngoscope reprocessing methods were evaluated, including the two most common methods used in community practice. The UV-C system offers the least costly approach to reprocessing in a high-volume clinic. However, Revital-Ox is more cost-effective in a low-volume clinic. Conclusions are limited by the fact that 83% of physicians report incomplete adherence with manufacturer recommendations and implicit costs associated with scope repair and replacement and patient safety could not be included in the model. We would recommend that guidelines for best practices in cleaning, disinfection, and sterilization, be revised to include recommendations specific to nasopharyngoscopes to avoid over-processing and improve adherence to guidelines. Billing schedules should be amended to include tray fees which will offset the relative burden associated with nasopharyngoscopy for physicians not practicing in hospital-based settings and ensure that community physicians continue providing this essential aspect of otolaryngology practice.

## Data Availability

The datasets used and/or analyzed during the current study are available from the corresponding author on reasonable request.
